# Balance and physical activity in teenagers and young adults with cochlear implants: a Swedish cohort study

**DOI:** 10.1186/s12887-026-06669-x

**Published:** 2026-03-05

**Authors:** Susanne Gripenberg, Luca Verrecchia, Cecilia Lidbeck

**Affiliations:** 1 ENT Unit Department of Clinical Science, Intervention and Technology, Karolinska Institutet, Stockholm, Sweden; 2 Medical Unit Allied Health Professional, Occupational Therapy and Physiotherapy, Karolinska University Hospital, Stockholm, Sweden; 3 Medical Unit Ear, Nose, Throat, Hearing and Balance, Karolinska University Hospital, Stockholm, Sweden; 4 Department of Women’s and Children’s Health, Division of Paediatric Neurology, Karolinska Institutet, Stockholm, Sweden; 5 Paediatric Neurology, Motion Analysis Laboratory, Astrid Lindgren Children’s Hospital, Karolinska University Hospital, Stockholm, Sweden

**Keywords:** Bruininks-Oseretsky Test of Motor Proficiency, Children, Kids Balance Evaluation Systems Test, Motor Proficiency, TAYACI, Vestibular Function, Vestibular Loss, Video Head Impulse Test

## Abstract

**Background:**

Vestibular function is a key component of balance and motor control, together with hearing for spatial orientation. Vestibular impairment is often present in case of childhood deafness, which is frequently resolved with cochlear implant (CI). With this study, we aimed to explore (1) the relation between balance, physical activity and vestibular function in teenagers and young adults since infancy (TAYACI), (2) the influence on balance with CI on vs. off, and (3) balance tests’ possibility to screen vestibular impairment in paediatric standard care.

**Methods:**

41 TAYACI, aged 12–22, and 20 normal hearing (NH) peers participated. Vestibular function was assessed with Video Head Impulse Test (vHIT). Balance was tested with the Bruininks-Oseretsky Test of Motor Proficiency (BOT-2) balance subtest, Kids Balance Evaluation Systems Test (Kids-BESTest) sections Sensory Orientation and Reactive Postural Responses, and a test of walking 10 m with head turns (10mW). Self-reported questionnaires were used to report experience of balance and physical activity.

**Results:**

vHIT ascertained vestibular impairment in 49% of TAYACI; balance was inferior in TAYACI compared to NH with lower scores on BOT2 subtest: mean (sd) 8.3 (4.0) vs. 15.1 (4.0) (*p* < 0.001), and on Kids-BESTest section Sensory Orientation: median (min, max) 13 (13–15) vs. 15 (15–15) (*p* < 0.001). Screening properties for vestibular impairment were found for BOT-2 balance subtest (AUC = 0.947; best score cut off: 9/25 – sensitivity: 100%, specificity: 89.7%), and singular items, especially BOT9 (standing on one leg on a balance beam with eyes closed) (AUC = 0.92, best cut off: 2.8/10 seconds – sensitivity:100% specificity:74%). None of the tests could distinguish between subjects with bilateral vestibular impairment or unilateral vestibular impairment, nor between TAYACI and NH with normal vestibular function. The CI activity (on/off) did not affect balance tests. 40% of TAYACI reported inferior balance than NH peers, but they were equally physically active.

**Conclusion:**

vestibular impairment is prevalent in TAYACI and represents an important determinant for balance skills. Standing on one leg on a balance beam with eyes closed is an accessible test to screen for vestibular impairment in children. Physical activity was comparable between TAYACI and NH peers.

**Trial registration:**

NCT 07079488.

**Supplementary Information:**

The online version contains supplementary material available at 10.1186/s12887-026-06669-x.

## Background

The function of the inner ear’s balance organ, known as vestibular function, is crucial for maintaining an upright position, stabilising gaze during head and body movements, and responding to falls. Balance and vestibular function play an essential role in fundamental motor functions such as standing and walking, of importance for physical activity and health. There is accumulated evidence that vestibular impairment contributes independently to disturbances in postural control such as spatial orientation and fall protection [1–3], leading to delay in reaching motor milestones in young children [4–6] and below-average performance on tests of motor skills by school age [7, 8].

In children with hearing impairment, up to 85% of children have been reported affected by vestibular impairment [9]. A cochlear implant (CI) is an implantable electronic hearing device that directly stimulates the auditory nerve endings through the electrical activity of an electrode placed in the cochlea. CI has been used to enable comprehension of speech in normal social environments in children with severe to profound hearing impairment since the early 2000s. Whether CI has a potentially detrimental effect on vestibular function is still debated [10–12]. An ongoing line of research has claimed that near-field electrical CI stimulation can influence vestibular balance organ activity. Therefore, functional balance capacity in children with CI on versus off has been studied, with contradictory results ranging from partially worse [13] to no difference [14, 15] and better [16, 17]. Furthermore, possible contributions on balance of spatial orientation by hearing cues have been claimed [18].

Standing or walking on soft or uneven surfaces, walking with quick head turns, fall reactions and visual dependency, are motor activities and functions that can be influenced in children with vestibular impairment. Some of these difficulties have been observed during clinical tests of motor proficiency, such as with the Bruininks-Oseretsky Test of Motor Proficiency – Second Edition (BOT-2) [19]. Results from the test have shown a certain degree of accuracy in detecting vestibular impairment in children with hearing impairment [16, 20].

The long-term impact of CI on functional balance and the impact on daily life activities such as physical activity in teenagers and young adults (T&Y) has, to our knowledge, not been explored. Identifying potential consequences for balance and daily life activities in this group may lead to a better understanding and enable individualised interventions. This study is part of the Teenagers and Young Adults with Cochlear Implants (TAYACI) project, which aimed to explore multidisciplinary long-term effects of paediatric cochlear implantation in adolescents according to language, cognition, hearing, balance, self-efficacy and Health Related Quality of Life (HRQoL) [21]. In particular, this study aimed to explore the relationship between vestibular function and (a) balance, (b) self-reported experience of balance and (c) physical activity in a TAYACI cohort and relate to normal hearing peers. The aim was also to explore the impact of CI presence and activity (on/off) on balance capacity in TAYACI. Finally, in case of a positive relation between performance on balance tests and vestibular impairment, to explore the accuracy of tests/ separate items as possible clinical screening tools for vestibular impairment.

## Methods

### Participants

All TAYACI operated at the Hearing and Implant Centre, Karolinska University Hospital, Sweden, were asked to participate in this cross-sectional, national cohort project [21].

Inclusion criteria were hearing impairment, implantation with CI before 30 months of age, age between 12 and 22 years at inclusion, and a standard school curriculum. All except for four participants, had bilateral cochlear implants. In one participant, there was a bimodal stimulation. For the other three participants with unilateral implants, it had not been possible with implantation despite profound hearing loss. Among the TAYACI, we expect a wide variation in vestibular function, truly affecting the balance performance. However, all these participants also have CI, and to understand the independent contribution of CI on balance, we have added a reference group with 20 age-matched T&Y with normal hearing (NH). All examinations were performed during 2022–2023.

All children/parents provided verbal and written consent before inclusion in the study. The study was conducted according to the Declaration of Helsinki and approved by the Swedish Ethical Review Authority (Dnr: 2021–04345 and the updated Dnr: 2025-05972-02).

### Instruments and procedure

All assessments were performed on the same day using a standardised protocol, starting with a test for vestibular function, followed by tests of balance capacity, and concluded with the completion of questionnaires.

#### Vestibular function

##### Video Head Impulse test (vHIT)

 [22] measures the function of the semicircular canals. The semicircular canals control the Vestibulo-ocular reflex (VOR), a motor response that functions to maintain a stable gaze during head movements. A parameter indicating the integrity of VOR is the gain between eye velocity and head velocity during quick passive head turns. Inner ear disorders can affect the VOR of one or both sides. The vHIT can measure the gain of both sides independently, and it represents the clinical standard for evaluation of vestibular impairment [23, 24]. Accordingly, a gain between 0.8 and 1.2 is defined as normal vestibular function for each side tested. A gain between 0.6 and 0.8 can be considered borderline. We therefore look at the incidence (> 50%) of the so-called refixation saccades, which consequently follow a defective VOR. A gain less than 0.6 is defined here as a vestibular impairment.

By these assumptions, vestibular function in each participant could be classified as having (1) normal vestibular function, (2) unilateral vestibular impairment (UVI) or (3) bilateral vestibular impairment (BVI).

vHIT was performed by a medical doctor with the ICS Natus Impulse device, with the patient sitting in a chair placed one meter from a target, wearing goggles that tracked and measured head and eye movements. Highly impulsive passive horizontal head rotations were conducted while the patient was instructed to maintain their gaze on the target in front of them. The testing took about 10 min.

#### Balance

##### The Bruininks-Oseretsky Test of Motor Proficiency (BOT-2)

 [19] is a norm-referenced test of motor proficiency, i.e. the ability to perform coordinated and efficient movements that involve fine and gross motor skills, consisting of eight subtests giving valid and reliable results for children and young adults aged 4–21 years. The test has been, for instance, used to follow motor proficiency in individuals with neurological disorders and to screen children with deficits in motor ability. In this study, we used the balance subtest consisting of nine items to assess dynamic and static balance in standing and walking. The items include tasks aiming to challenge balance with different bases of support and with or without vision, such as standing on one leg with eyes open or closed (Table 1). The capacity to perform the task is scored from 0 to 4 (5 in one item) giving a point score, derived from raw scores, i.e. time in seconds to maintain a position according to detailed descriptions. Point scores are normalised on age-normative data, becoming “scale” scores ranging from 0 to 25 points. Scale scores are classified in five categories and compared to average; (1) Well below average (5 or less), (2) Below average (6–10), (3) Average (11–19), (4) Above average (20–24) and (5) Well above average (25).

**Table 1:** Separate items, Subtest/ Sections from balance instruments

Tests	BOT-2	Kids-BESTest		Walking 10 m with and without head turns
Subtest/ Sections	Balance Subtest	Reactive Postural Responses	Sensory Orientation	
Items	1. Standing with feet apart on a line, eyes open	14. In place response-forward	19a. Standing with feet together on firm surface, eyes open	1. Walking 10-m. Self-selected speed, no head turns
	2. Walking forward on a line	15. In place response-backward	19b. Standing with feet together on firm surface, eyes closed	2. Walking 10-m. Self-selected speed, head turns
	3. Standing on one leg on a line, eyes open	16. Compensatory stepping correction-forward	19c. Standing with feet together on foam surface, eyes open	3. Walking 10-m. Fast as possible, head turns
	4. Standing with feet apart on a line, eyes closed	17. Compensatory stepping correction-backward	19d. Standing with feet together on foam surface, eyes closed	
	5. Walking forward heel-to-toe on a line	18. Compensatory stepping correction-lateral	20. standing shoulder with apart on an incline ramp, eyes closed	
	6. Standing on one leg on a line, eyes closed			
	7. Standing on one leg on a balance beam, eyes open			
	8. Standing heel-to-toe on a balance beam, eyes open			
	9. Standing on one leg on a balance beam, eyes closed			

Short descriptions of separate items, subtest, and sections adapted from Bruininks-Oseretsky Test of Motor Proficiency Second Edition (BOT-2), Kids-Balance Evaluation System Test (Kids-BESTest) and Walking 10 m with and without head turns, used as test of balance.

In this study, we analysed the scale scores from the balance subtest as a measure of balance capacity. In addition, we have separately analysed the raw scores (in seconds) for each of the seven separate items measuring static balance, aiming to identify the most indicative item of vestibular impairment. Given that these scores are not normed, the analyses were conducted compared to raw scores in the reference group.

##### Kids-Balance Evaluation System Test (Kids-BESTest)

 [25] is an assessment designed to identify and classify postural control deficits, adjusted for children from the adult BESTest developed by Horak et al. [26]. BESTest was developed for older people, neurological conditions, vestibular and cognitive disorders. Kids-BESTest has been used in children with typical development and ambulant children with CP, giving valid and reliable results [27], but to our knowledge, has not been used in children with vestibular impairment or hearing impairment. In this study, two out of six sections were used. The one, Reactive Postural Responses, consists of six items evaluating balance reactions like “in place response” forward and backward, and “compensatory stepping correction” forward, backward, and lateral. How the participant performs the task is scored (0–3). The total is 18 points. The other, Sensory Orientation, consists of five items evaluating the impact of standing on different surfaces with eyes open or closed (Table 1). The number of seconds the participant managed to stand is recorded and gives a point scale (0–3). The total is 15 points. In both sections, higher points indicate a better balance. In this study, both the total points and the timed performance on all separate items were used as potential markers of vestibular function.

##### Walking 10 m with and without head turns

 [28] is a test where the participant first walks ten meters at self-selected speed, then walks again and turns their head to the left and right side in connection with each step. Difference in time in seconds and number of steps (frequency) is the outcome, which has been demonstrated to be related to vestibular impairment in adults with acute unilateral vestibular impairment, showing reduced walking speed compared to a reference group. In this study, we added the item “to walk as fast as possible without running” while walking with head turns (Table 1). According to our clinical experience, the difference would be much more evident at higher speeds. We also added a scale (0–3), based on a scale used in Kids-BESTest -section stability in gait, to note the impact on balance (quality), where higher points indicate a better balance.

#### CI on and off

Eight tasks of balance (items 4, 6 and 9 from subtest balance BOT-2, and items 14, 15, 19b and 19d from Kids-BESTest and item 4 from Walking 10 m with and without head turns) were tested in two conditions: (i) with CI turned on and (ii) CI off in TAYACI, or with headphones masking white noise to simulate in NH the sound deprivation as in TAYACI with CI off. The eight tasks chosen were expected to be the most balance challenging according to own clinical experience and previous research [16].

All tests of balance were conducted by the same experienced physiotherapist, who was blinded to results from vHIT and medical information. The testing procedure took approximately 40–50 min.

#### Self-assessment of physical activity

##### Saltin-Grimby Physical Activity Level Scale (SGPALS)

 [29, 30] is a four-level questionnaire to assess the level of physical activity in leisure time, through the question “How much do you move and exert yourself physically during your leisure time?”. The question refers to the past year [see Additional file 1].

#### Self-reported questions

A questionnaire comprising seven questions on experience of physical activity, balance, participation in physical education in school and motor development, constructed by the authors in collaboration with local expertise. All participants, either alone or assisted by parents, filled out the questionnaires. Three of the questions were multiple-choice, and four were free text [see Additional file 2].

### Data analysis and statistics

Statistical analyses were conducted with IBM SPSS Statistics version 28.01.1 [14].

Descriptive data are presented as numbers, frequencies, percentages, means (standard deviation) or medians (25%-75%).

Between-group analyses were performed according to different clinical determinants: (1) presence of CI: TAYACI vs. NH, (2) presence of normal vestibular function regardless of the presence of CI: total sample with Normal Vestibular Function (NVF) vs. TAYACI with vestibular impairment (unilateral and bilateral) (VL), (3) classification of vestibular function in subgroups: TAYACI with normal vestibular function (CI-NVF), TAYACI with unilateral vestibular impairment (CI-UVI), TAYACI with bilateral vestibular impairment (CI-BVI) and NH (all showing normal vestibular function).

To determine possible differences in balance capacity (BOT-2, Kids BEST-test, Walking 10-m with and without head turns), between NH and TAYACI, respectively NVF and VL (two-groups), t-test or Mann-Whitney U test were used. To analyse possible differences on balance tests between the vestibular subgroups (more than two groups), Kruskal-Wallis H test, with Bonferroni corrections for multiple comparisons was used. Kruskal-Wallis H test was also used to analyse physical activity level (SGPALS), self-reported participation in physical education and experience of balance in all groups.

To explore possible differences in self-reported spare-time activities and experience of physical education between TAYACI and NH, a Chi-Square test was used.

To determine the diagnostic accuracy of balance tests, or the items separately in detecting vestibular impairment, a ROC curve analysis was used. Finally, to explore the effect of CI status on/off on balance in TAYACI, respectively white noise on/off in the NH, within the groups, Wilcoxon Signed Rank test and Two-way mixed ANOVA were used.

Significance level for univariate analyses was *p* < 0.05 and *p* < 0.0083 for multivariate analyses.

## Results

### Participants

Forty-one of the 50 invited TAYACI consented to participate (23 females, 18 males; median age 17 years, range 12.8–22.2). Test of vestibular function was completed by 37, the balance tests and SGPALS by 41, and the patient-reported questions by 39 TAYACI. The number of participants varied due to logistical and organisational issues. Participants were living in different regions and were engaged in a complex, multi-professional investigation with multiple operators involved. Twenty NH (10 females, 10 males; median age 15.4 years, range 12.7–21.6) constituted a reference group. All 20 NH performed all tests and questionnaires.

Age distribution of the 37 TAYACI with all tests performed in the present study, differed from the originally 50 invited TAYACI, who were 1.2 years older (*p* = 0.04), while gender distribution did not differ significantly.

### Vestibular function

vHIT ascertained vestibular impairment in 18/37 (49%) of TAYACI, divided in BVI in 13 (35%) and UVI in 5 (14%), whereas normal vestibular function was found in 19 (51%). All NH had normal vestibular function.

### Balance

#### BOT-2, Balance subtest

Scale scores.

1. TAYACI vs. NH

Scale scores of BOT-2 indicated difficulties with balance in standing and walking in TAYACI compared to NH, with significantly lower mean (sd) scale scores: 8.3(4.0) vs. 15.1(4.0); t=-6.24, (*p* < 0.001).

2) NVF vs VL

**Fig. 1:**
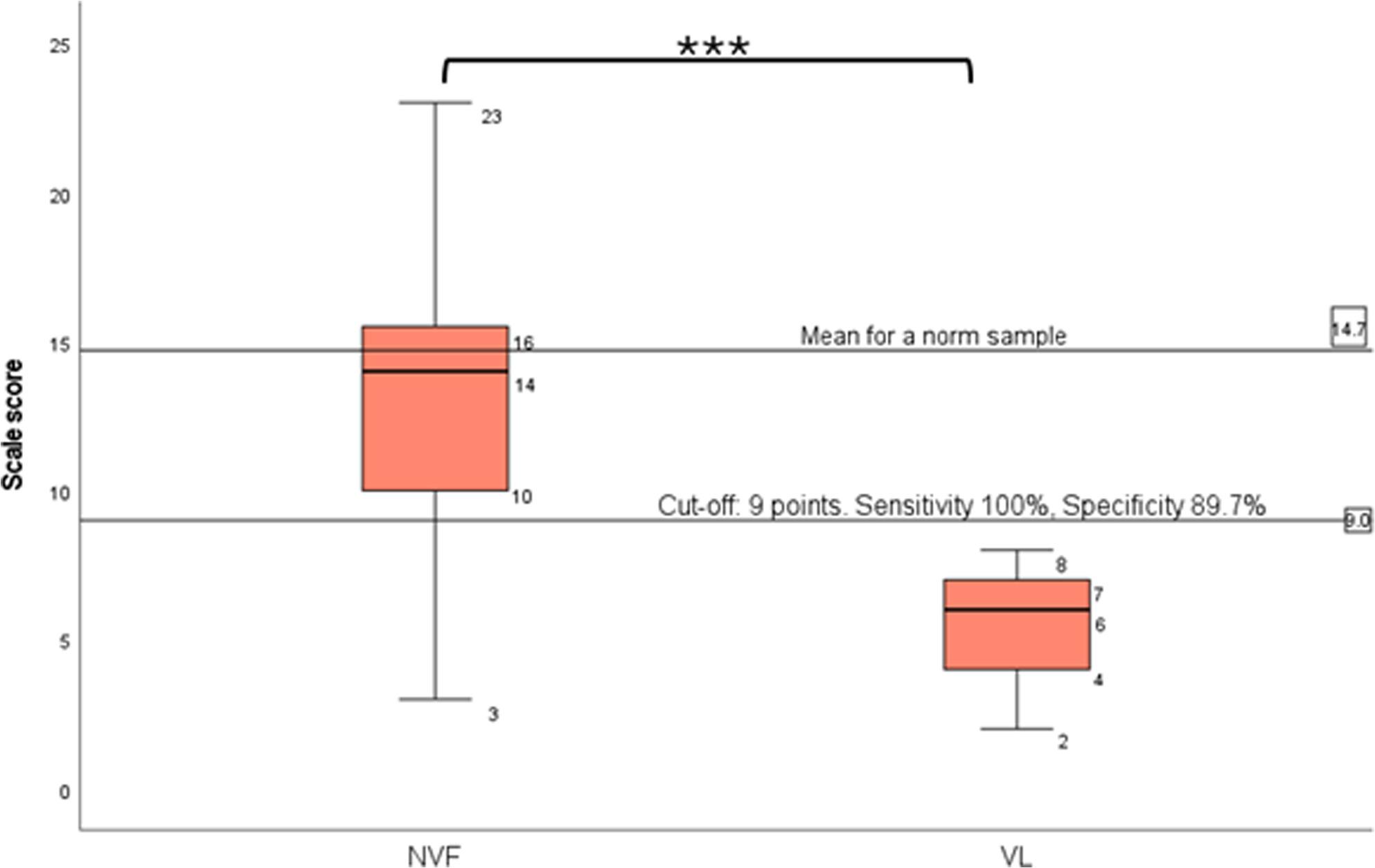
Balance from the subtest balance in Bruininks-Oseretsky Test of Motor Proficiency (BOT-2) in teenagers and young adults with normal vestibular function (NVF), n = 39, and with vestibular impairment (VL), n = 18. Data are presented as median, min, max, 25% percentile and 75% percentile scale scores. The horizontal line represents a cut-off score of 9/25 points, the best level to detect a vestibular impairment, with a sensitivity of 100% and specificity of 89.7% (p = 0.000)

Scale scores of BOT-2 indicated inferior balance in participants with vestibular impairment (all TAYACI). VL compared to NVF (19 TAYACI and 20 NH) had significantly lower scale scores: 5.6(1.6) vs. 13.1(4.4); t = 9.3 (*p* < 0.001) [see Additional file 3]. Furthermore, to separate VL from NVF a ROC curve analysis yielded an AUC of 0.947 (*p* = 0.000), and 9/25 was identified as the optimal cut-off in score, with a sensitivity of 100% and specificity of 89.7% (Fig. 1).

3) Vestibular subgroups

In further subgroup analysis with respect to vestibular function (CI-NVF, CI-UVI, CI-BVI and NH, respectively), the distribution of scale scores from BOT-2 was significantly different; H (3) = 34.286 (*p* < 0.001). Post-hoc analysis indicated inferior balance in CI-UVI compared to NH (*p* < 0.01), and inferior balance in CI-BVI compared to NH (*p* < 0.001) and CI-NVF (*p* < 0.01) [see Additional file 3].

Overall, this analysis of scale scores showed that capacity on the balance tests differed between TAYACI and NH, but the difference was attributable to the presence or absence of a normal vestibular function. This can be confirmed by the vestibular subgroup analysis, which showed that a normal vestibular function was superior to belonging to TAYACI vs. NH and CI-UVI vs. CI-BVI regarding capacity on the tests. In a few words, balance was a matter of vestibular function.

##### Separate items, static balance within the balance subtest

*1) TAYACI vs. NH and 2) NVF vs. VL*

Analysis of raw scores on separate items measuring static balance within the balance subtest from BOT-2, showed the same pattern, i.e. the presence of vestibular function was the only determining factor in defining the performance. In 5 of 7 of the items, raw scores indicated inferior balance with significantly shorter times in seconds in TAYACI compared to NH, and in VL compared to NVF on items 4, 6, 7, 8, and 9 (*p* < 0.001), respectively [see Additional file 4].

**Fig. 2:**
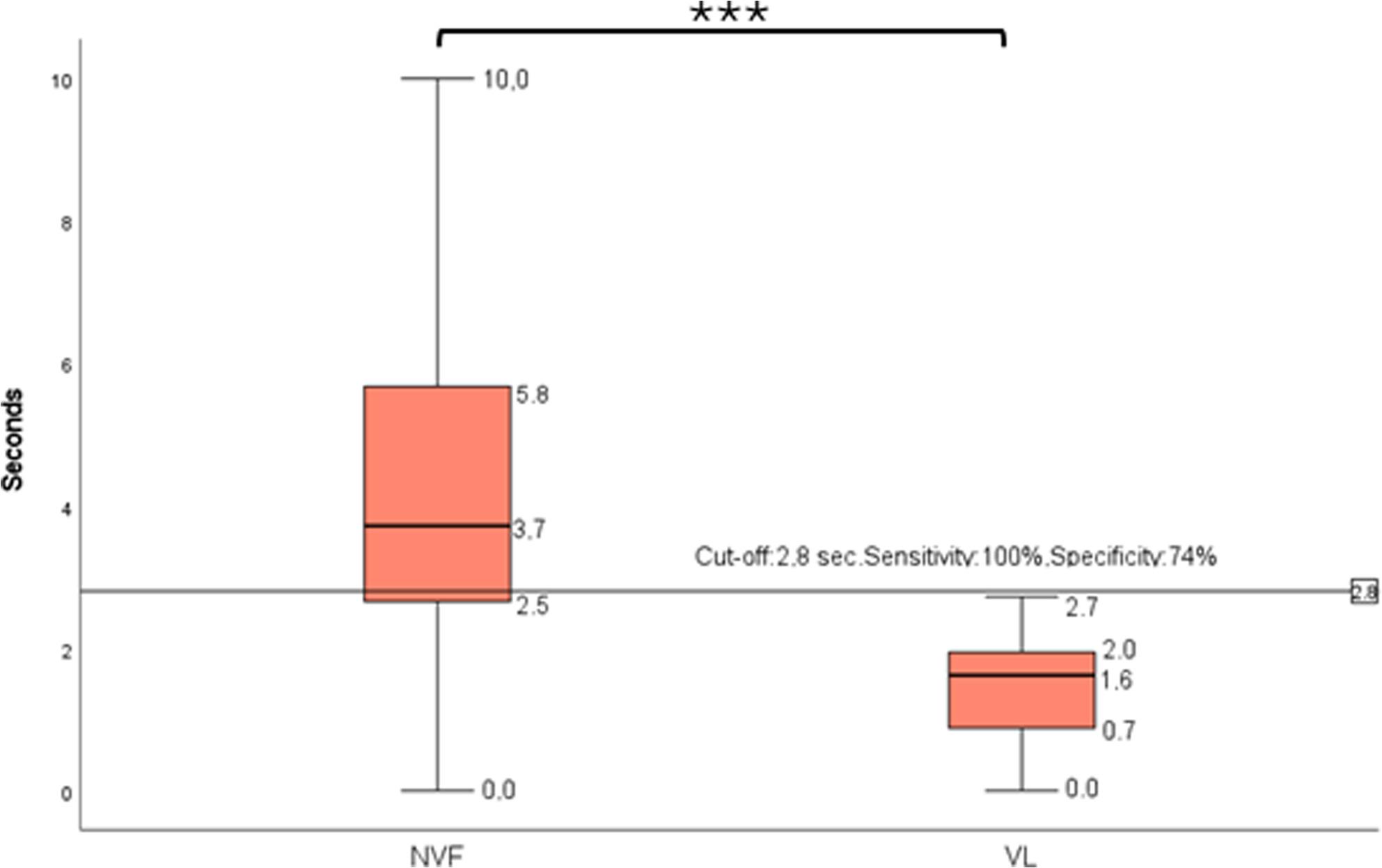
Balance measured with item BOT9, standing on one leg on a balance beam with eyes closed, from Bruininks-Oseretsky Test of Motor Proficiency (BOT-2) in teenagers and young adults with normal vestibular function (NVF), n = 39, and with vestibular impairment (VL), n = 18. The horizontal line represents a cut-off of 2.8 s, the best level to detect a vestibular loss, with a sensitivity of 100% and specificity of 74% (p = 0.000)

Looking at the screening ability of the separate items in detecting vestibular impairment, a ROC Curve analysis identified 3 BOT-2 items: item 4 (AUC = 0.88), item 6 (AUC = 0.9) and item 9 (AUC = 0.92) (Table 2). Item 9 ranked highest with the best level of sensitivity of 100% and specificity of 74% for vestibular impairment, reached with a cut-off of 2.8/10 seconds (*p* = 0.000) (Fig. 2).

**Table 2:** AUC, Cut-off values, Sensitivity and Specificity for separate items from BOT-2 and Kids-BESTest, for identifying vestibular impairment

Separate items	AUC	Best cut-off (sec)	Sensitivity (%)	Specificity (%)
BOT3	0.64	-	-	-
BOT4	0.88	8.0	83	92
BOT6	0.90	6.6	100	67
BOT7	0.77	-	-	-
BOT8	0.74	-	-	-
BOT9	0.92	2.8	100	74
Kids-BESTest 19d	0.91	7.1	76	95

Area under the curve (AUC), best cut-off value in seconds for screening VL from NVF. Values under cut-off indicating vestibular impairment. Sensitivity and Specificity in % for separate items from Bruininks-Oseretsky Test of Motor Proficiency (BOT-2) and Kids-Balance Evaluation System Test (Kids-BESTest). Items with an AUC of more than 0.8 indicate clinical relevance and are highlighted in bold text.

*3) Vestibular subgroups*

In additional vestibular subgroup analysis of separate items from BOT-2, all subgroups managed to stand significantly shorter time compared to NH, on item 3 (*p* < 0.01), and items 4, 6, 7, 8 and 9 (*p* < 0.001), indicating inferior balance in the subgroups. Post-Hoc test showed a general pattern in all these items where none of the items could differentiate CI-UVI from CI-BVI, or CI-NVF from NH [see Additional file 4].

#### Kids-BESTest

##### Section Reactive Postural Responses

Total points.

In total median points from Reactive Postural Responses, there was no statistically significant difference between any of the groups or subgroups [see Additional file 3].

Point scores on separate items.

None of the points on the separate items showed a statistically significant difference in any of the groups or subgroups [see Additional file 5].

##### Section sensory orientation

Total points.

1. TAYACI vs. NH

Total median points from Section Sensory Orientation indicated difficulties with balance in standing in TAYACI compared to NH, with significantly lower median points (*p* < 0.0012).

2) NVF vs. VL

Total median points from Section Sensory Orientation were significantly lower in VL compared to NVF (*p* < 0.001) [see Additional file 3].

3) Vestibular subgroups

In further vestibular subgroup analysis, the distribution of points from Section Sensory Orientation was significantly different between the subgroups H (3) = 31.192 (*p* < 0.001). Post-Hoc analysis revealed more difficulties with balance in both CI-UVI (*p* < 0.05) and CI-BVI (*p* < 0.001) compared to NH. More difficulties were also found in CI-BVI compared to CI-NVF (*p* < 0.001) see Additional file 3].

Concluding, Kids-BESTest gave the same indications as BOT-2, i.e. the presence of normal vestibular function was the main determinant of balance capacity.

Point scores and raw scores on separate items.

Results from all separate items from Section Sensory Orientation, except for item 19d (standing with feet together on foam with eyes closed) showed a ceiling effect in all groups and most subgroups, leading to the decision to discard these items from further analysis. Analysis of both point scores and raw scores (time in seconds) related to 19d was conducted.

1. TAYACI vs. NH

In TAYACI vs. NH, time in seconds on item 19d was significantly shorter, 27(4.6–30) vs. 30(30–30) (*p* < 0.001) and point scores were lower, 1 (1–3) vs. 3(3–3) (*p* < 0.001).

2) NVF vs. VL

VL could stand for a shorter time on item 19d compared to NVF 4.6(2.7–7.4) vs. 30(30–30) (*p* < 0.001) and point scores were lower, 1(1–1) vs. 3(3–3) (*p* < 0.001), indicating impaired balance.

A ROC Curve analysis yielded an AUC of 0.91, but we considered item 19d not clinically suitable for vestibular impairment screening since the best sensitivity was 76.5%.

3) Vestibular subgroups

Results from vestibular subgroup analysis of item 19d revealed significantly different distribution in times in seconds in standing between the groups, H (3) = 34.841 (*p* < 0.001). Post-hoc analysis indicated inferior balance in CI-UVI (*p* < 0.01) and CI-BVI (*p* < 0.001) compared to NH, and CI-UVI (*p* < 0.05) and CI-BVI (*p* < 0.001) compared to CI-NVF.

The distribution in median points for item 19d was also significantly different between the subgroups, H (3) = 32.692 (*p* < 0.001). CI-UVI (*p* < 0.05) and CI-BVI (*p* < 0.001) showed significantly lower scores compared to NH, and CI-BVI (*p* < 0.001) showed lower scores compared to CI-NVF [see Additional file 5].

##### Walking 10-meters with and without head turns

*1)TAYACI vs. NH and 2) NVF vs. VL*

Walking 10-m, Self-selected speed, head turns, points, was the only test condition that showed a statistically significant difference between TAYACI and NH and between NVF and VL. A lower median point was found in TAYACI compared to NH: 3(2–3) vs. 3(3–3) (*p* < 0.05), and a lower median point was found in VL compared to NVF: 2(1.8-3) vs. 3(3–3) (*p* < 0.001).

*3)Vestibular subgroups*

In further vestibular subgroup analysis, the distribution in points was significantly different in Walking 10-m, Self-selected speed, head turns, points H (3) = 19.243(*p* < 0.001), where CI-BVI showed significantly lower points compared to NH (*p* < 0.01) and CI-NVF (*p* < 0.01).

The distribution in seconds was significantly different in Walking 10-m, Self-selected speed, no head turns, seconds H (3) = 9.489(*p* = 0.023), where CI-UVI showed significantly lower time in seconds compared to NH (*p* < 0.05) [see Additional file 3].

##### CI turned on/off

We chose to analyse the role of CI on/off, with respect to group belonging, on the four items which had the best diagnostic precision in detecting vestibular impairment (BOT-2 items 4, 6, 9, and Kids BESTest 19d).

1. TAYACI vs. NH

Scores, time in seconds, regarding TAYACI vs. NH showed a general ceiling effect in NH in items 4, 6, and 19d in both conditions CI on/off. We interpreted this pattern as an off-scale distribution of data not useful for study purposes, and for this reason discarded from further analysis. We have thus limited the analysis to only TAYACI with CI on/off conditions, resulting in no significant difference in results (Wilcoxon Signed Rank test; item 4: *p* = 0.473, item 6: *p* = 0.519 and item 19d: *p* = 0.520). Regarding item 9, a two-way mixed ANOVA showed no statistically significant interaction between group belonging and CI condition; F(1, 59) = 0.69, *p* = 0.79, partial η2 = 0.001. The main effect of CI-condition showed no statistical difference within groups; F(1, 59) = 0.107, *p* = 0.75, partial η2 = 0.002, whereas the main effect of group belonging showed a statistically significant difference between the groups; F(1, 59) = 49.6, *p* < 0.001, partial η2 = 0.46 [see Additional file 6].

2) NVF vs. VL

In analysis of VL vs. NVF, a ceiling effect was observed in NVF in items 4 and 19d. The analysis was therefore restricted to the effect of CI on/off in only VL, finding a significant difference in distribution for item 19d (*p* < 0.001) but not for item 4 (*p* = 0.705). For items 6 and 9, there was no significant interaction effect between group belonging and CI condition, item 6; F(1, 55) = 2.79, *p* = 0.1, partial η2 = 0.48, and item 9; F(1, 55) = 0.064, *p* = 0.8, partial η2 = 0.01. The main effect of CI-condition showed no statistical difference for item 6; F(1, 55) = 0.36, *p* = 0.55, partial η2 = 0.006, nor for item 9; F(1, 55) = 0.018, *p* = 0.89, partial η2 = 0.000. The main effect of group belonging for items 6 and 9 was statistically significant; 6; F(1, 55) = 63.1, *p* < 0.01, partial η2 = 0.934, 9; F(1, 55) = 26.9, *p* < 0.001, partial η2 = 0.329 [see Additional file 7].

3) Vestibular subgroups

In additional vestibular subgroup analysis, both items 4, 6 and 19d showed ceiling effect in one or more subgroups, and further analysis was therefore not performed. Regarding item 9, there was no interaction effect between CI on/off and vestibular subgroups: F(3, 53) = 0.054, *p* = 0.98, partial η2 = 0.03. There was no main effect regarding CI condition; F(3, 53) = 0.007, *p* = 0.93, partial η2 = 0.000. However, group belonging showed a statistically significant main effect, F(3, 53) = 20.59, *p* < 0.001, partial η2 = 0.54 and Post Hoc comparisons revealed a statistically significant group difference only when comparing NH with all other vestibular subgroups. (*p* < 0.001) but not within the latter [see Additional file 8].

### Self-assessment of Physical activity

#### Saltin-Grimby Physical Activity Level Scale (SGPALS)

**Fig. 3:**
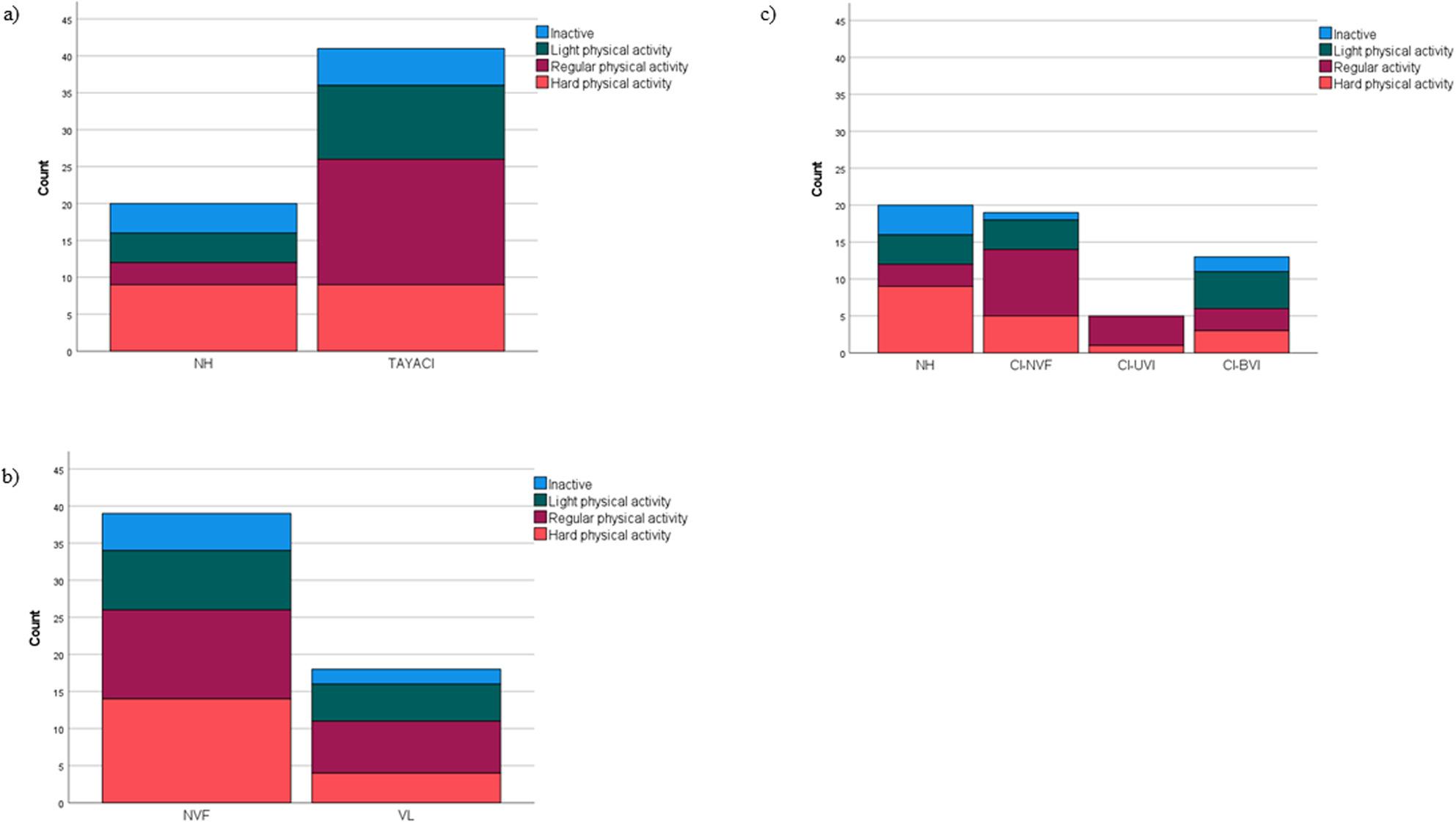
Self-reported physical activity according to Saltin-Grimby Physical Activity Level Scale (SGPALS). Distribution of levels in teenagers and young adults: **a**) with normal hearing (NH), n=20, and with cochlear implants (TAYACI), n=41, H(1)=0.389(p=0.533). **b** with normal vestibular function (NVF), n=39, and with cochlear implants and vestibular impairment (VL), n=18, H(1)=0.514(p=0.474). **c** in vestibular subgroups: with normal hearing (NH), n=20, with cochlear implants and normal vestibular function (CI-NVF), n=19, with cochlear implants and unilateral vestibular impairment (CI-UVI), n=5, with cochlear implants and bilateral vestibular impairment (CI-BVI), n=13, H(3) =1.827 (p=0.609)

In level of physical activity there was no statistically significant difference between TAYACI and NH, between NVF and VL, nor between Vestibular subgroups. The distribution of the activity levels is illustrated in Fig. 3.

#### Self-reported questions

1.“What activities do you perform in your spare time?” (Free text).

Ninety-seven per cent of TAYACI compared to 85% per cent of NH reported being physically active (*p* = 0.083). Being physically active was defined as having one or more physical activities like racket sports, other ball sports, walking, horse riding, weightlifting, etc.

3.“How often do you usually participate in physical education and health in school?”

**Fig. 4:**
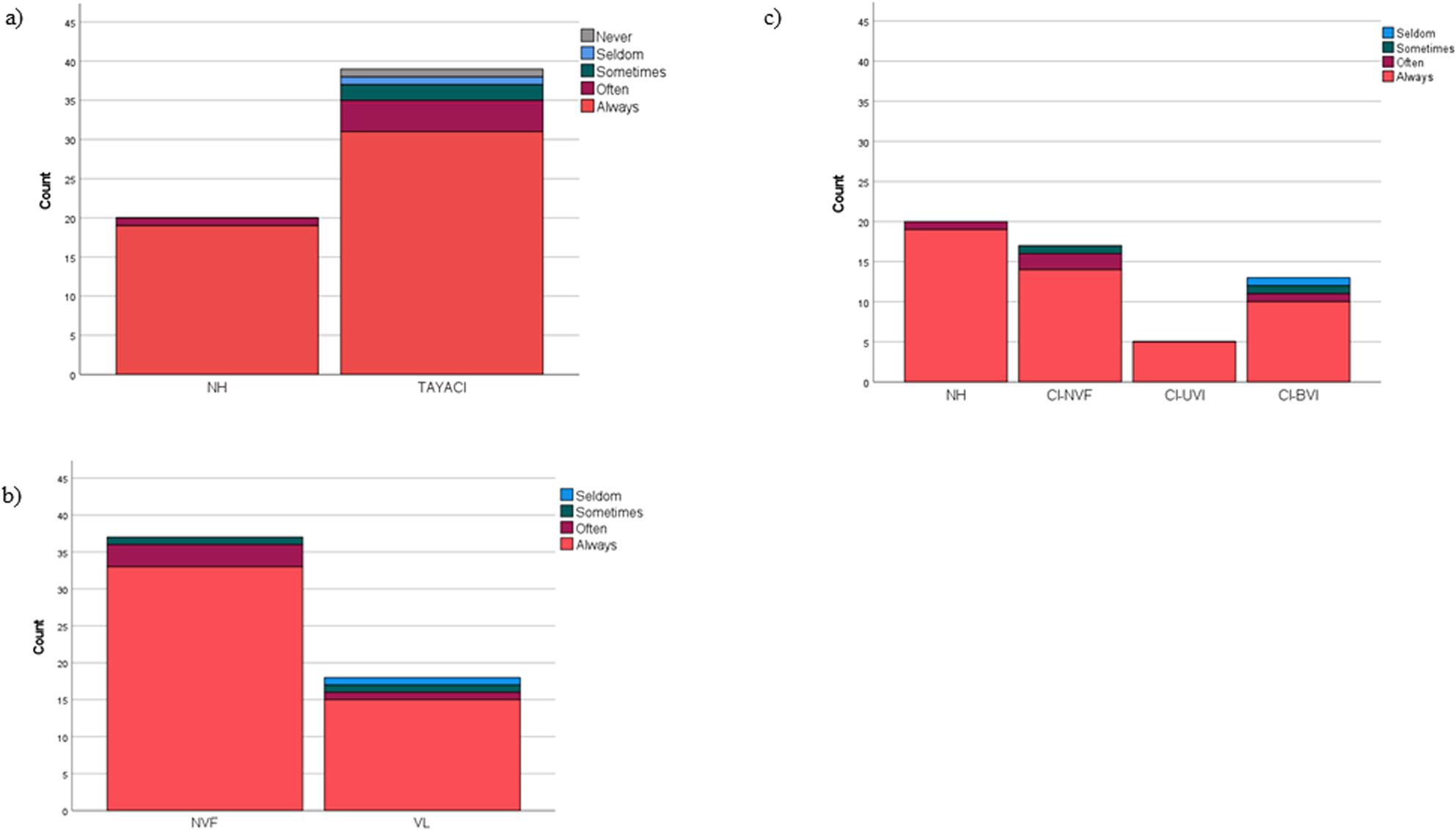
Self-reported participation in physical education and health in school from the self-reported questionnaire. Distribution of answers to the question “How often do you usually participate in physical education and health in school?” in teenagers and young adults: **a**) with normal hearing (NH), n=20, and with cochlear implants (TAYACI), n=39. H(1)=2.563(p=0.109). **b** with normal vestibular function (NVF), n=37, and with cochlear implants and vestibular impairments (VL), n=18. H(1)=0.486 (p=0.486). **c** in vestibular subgroups: with normal hearing (NH), n=20, with cochlear implants and normal vestibular function (CI-NVF), n=17, with cochlear implants and unilateral vestibular impairment (CI-UVI), n=5, and cochlear implants and bilateral vestibular impairment (CI-BVI), n=13. H(3) =3.586 (p=0.310).

There was no statistically significant difference in participation in physical education between TAYACI and NH, NVF and VL, nor between Vestibular subgroups (Fig. 4).

4.“Please describe if there is anything you avoid doing in physical education and health in school?” (Free text).

Fifty-seven per cent of TAYACI, compared to 55% of NH, reported that they did not avoid anything. The most common reason for avoidance in TAYACI was practical difficulties with the CI.

6. “How do you find your balance compared to others in your age?”

**Fig. 5:**
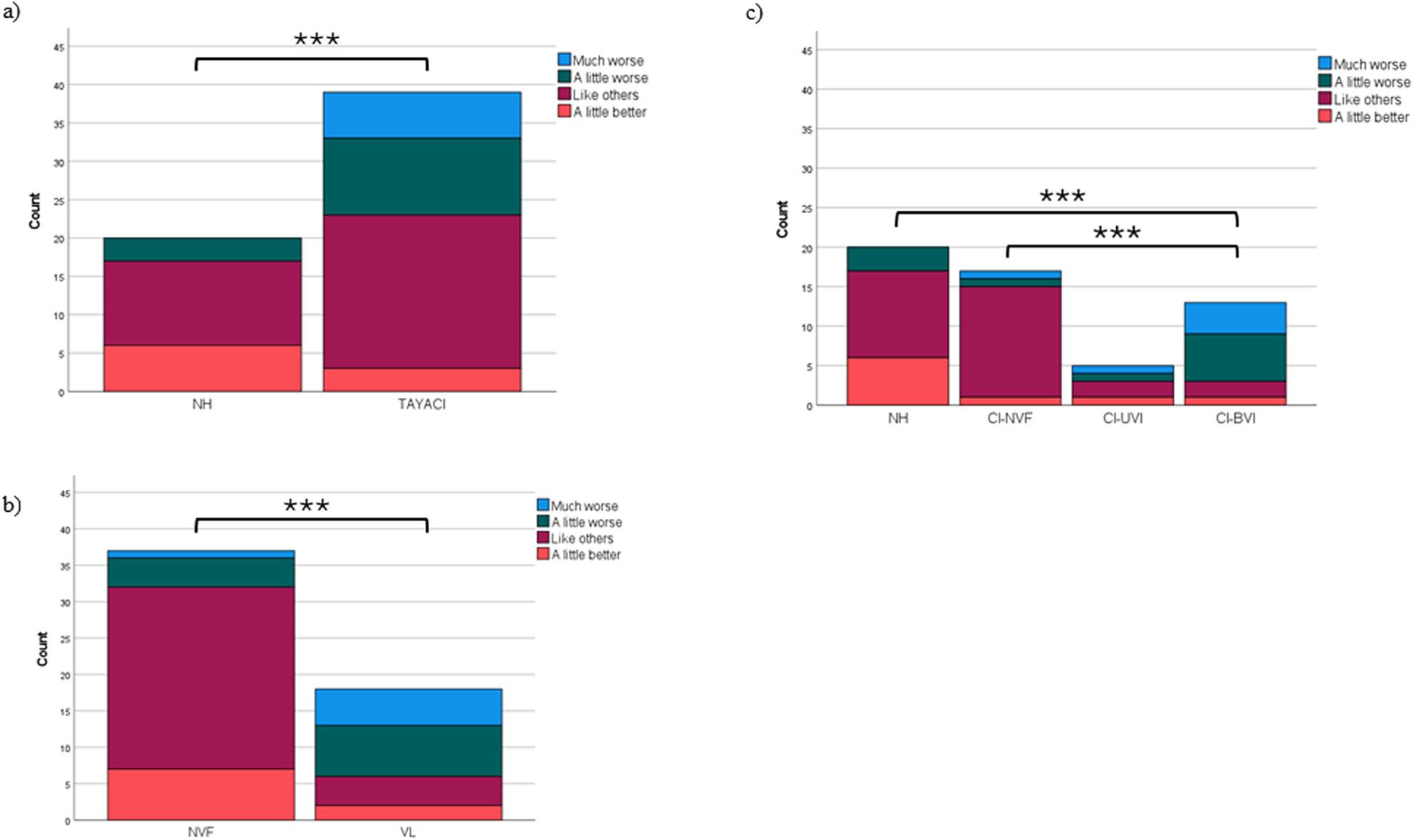
Self-reported experience of balance from the self-reported questionnaire. Distribution of answers to the question “How do you find your balance compared to others in your age?” in teenagers and young adults: **a**) with normal hearing (NH), n=20, and with cochlear implants (TAYACI), n=39. H(1)=7.240 (p=0.007). **b** with normal vestibular function (NVF), n=37, and with cochlear implants and vestibular impairment (VL), n=18. H(1)=11.398 (p<0.001). **c** in vestibular subgroups: with normal hearing (NH), n=20, with cochlear implants and normal vestibular function (CI-NVF), n=17, with cochlear implants and unilateral vestibular impairment (CI-UVI), n=5, and with cochlear implants and bilateral vestibular impairment (CI-BVI), n=13. H(3) =14.132 (p=0.003). Pairwise comparison showed a significant difference between CI-BVI and NH (p<0.001) and between CI-BVI and CI-NVF (p=0.006)

There was a statistically significant difference in distribution of experience of balance between TAYACI and NH; H (1) = 7.240 (*p* = 0.007), between NVF and VL; H (1) = 11.398 (*p* < 0.001) and also between Vestibular subgroups; H (3) = 14.132 (*p* = 0.003). The distribution of the experience is illustrated in Fig. 5. In vestibular subgroups, there was a significant difference in experience of balance where CI-BVI experienced inferior balance compared to NH (*p* < 0.001) and compared to CI-NVF (*p* = 0.006).

7. “Do you know if you learned any of the following late?” (according to riding a bike, walking and sitting).

It was sometimes difficult to answer this question for the participants who did not have their parents with them, and we therefore chose not to analyse the question.

## Discussion

This cohort study explored vestibular function, balance in standing and walking and physical activity in teenagers and young adults with CI (TAYACI) using objective and self-reported instruments. Results confirmed vestibular impairment in a high proportion of the participants as previously reported [9]. When balance was provoked, only those with vestibular impairment, regardless of whether unilateral or bilateral, and the presence and activity (on/off) of CI, showed difficulties with standing and walking during testing. However, TAYACI compensated for their functional constraints well and were as physically active as their peers with normal hearing. A set of easily accessible items for screening vestibular impairment in this group was proposed.

### Which factors affected balance in TAYACI?

In a study by De Kegel et al. [31], children with hearing impairment exhibited a decline in motor capacity, which was only partially reversed two years after cochlear implantation. The authors highlighted the importance of longer follow-ups to determine whether the children eventually catch up with their peers. In our long-term follow-up study, vestibular impairment in TAYACI had functional consequences on balance during a testing situation, more pronounced in CI-BVI. More specifically, CI-BVI had difficulties standing on a small base of support both with and without vision and managed to stand for a shorter time than CI-NVF and NH. This was evident on the BOT-2 balance subtest and Kids-BESTest sensory orientation. The results were consistent with those of Oyewumi et al. [20] when measuring children with CI and BVI using BOT-2. Children and youths with UVI showed significantly more difficulties in balance abilities compared to controls with normal hearing in a study by Cushing et al. [16], which aligned with our results. However, since the CI-UVI in our cohort were few (5/37 subjects), our material was not optimal to distinguish the balance capacity of subjects from this vestibular subgroup. However, it was clear for most of the tests that CI-UVI performed more like CI-BVI than other subgroups. Kids-BESTest has, to our knowledge, not been employed for testing T&Y with CI. However, Janky et al. [32] used Standing Balance Test (SBT) from the Modified NIH Toolbox, which contains comparable items to the Sensory Orientation Sect. [25]. They found that children with BVI and UVI had more difficulties with shorter time in standing on the floor or foam with closed eyes and standing on foam with open eyes compared to peers with NVF, which was in accordance with our findings.

### Which balance tests had a screening value in identifying vestibular impairment?

Our data showed that using the scale score for the Balance subtest (BOT-2) was an effective method for screening vestibular impairment in TAYACI, consistent with the findings of Oyewumi et al. [20]. Janky et al. reached a similar conclusion, but they used the total point score, which did not allow for comparison to a normative sample of the same age [32].

Since one of our aims in this study was to identify screening tests that are easy and accessible to administer in clinical settings, we analysed results from separate items of the tests BOT-2 and Kids-BESTtest, respectively. The results identified as the most suitable for screening vestibular impairment were items 4, 6, and 9 from BOT-2. All these items involved standing with eyes closed on a small base of support, which from clinical experience and previous research, is known to be challenging for children with vestibular impairment. Item 9, which we considered the most effective screening tool, implied standing on one leg on a balance beam with eyes closed. Standing less than 2.8 s was strongly indicative of vestibular impairment (sensitivity 100%, specificity 74%). Subjects in the age span of 12–21 years, performing less than 3 s on item 9 and in the absence of other explanations, could be subject to vestibular impairment and are to be addressed for vestibular in-depth evaluation. This was also noted by Cushing et al. [16], who found that the balance beam posed significant difficulty for children with CI when their eyes were closed. Oyewumi et al. [20] identified item 6 in BOT-2 as the best predictor of BVI in children. We concluded that it was the second-best item in our cohort.

Janky et al. concluded that a Single Leg Stance (SLS) with eyes closed for 30 s was a good functional test to predict vestibular impairment in children with CI, with a cut-off criterion of 5 s (sensitivity 88% and specificity 86%) [32]. In our study, we used BOT-2, item 6, which is a similar task except for the shorter time of 10 s required to get a maximum score. The shorter time may be a limitation of using item 6, as some participants with UVI or NVF could have been identified from a 30-second testing to avoid a ceiling effect and to measure changes in capacity, which was also concluded in a study by Condor and Cremin [33]. When selecting tests for this study, we chose standardised and preferably norm-referenced instruments developed to evaluate balance that are commonly used in a paediatric clinic. Results from the previous study indicated that a longer time in one leg standing, as with the SLS, could have also revealed a UVI in the present study exploring balance in young adults.

With the Kids-BESTest, a ceiling effect was observed across the different groups in all items except 19d (standing with feet together on foam with eyes closed). We concluded that the tasks in the test were not challenging enough to stratify differences in balance between the groups in our cohort. Although the AUC for 19d was 0.91%, its maximum sensitivity was 76.5%, so it was not optimal for screening purposes.

Our subgroup comparison revealed that none of the tests could be used to distinguish difficulties in performing balance provoking tasks between CI-UVI and CI-BVI, CI-UVI and CI-NVF, or CI-NVF and NH. While distinguishing between UVI and BVI or UVI and normal vestibular function would certainly be valuable in the clinic, we considered the ability to identify vestibular impairment from normal vestibular function to be the most powerful screening property. The differentiation between UVI and BVI can then be addressed through a more detailed in-depth assessment. Therefore, the analysis between NVF and VL could provide greater utility for caregivers and researchers when screening for balance issues, especially if achievable with a single item as BOT9 from BOT-2. In existing studies in the field, there were no subgroup comparisons, except for Janky et al., who reached a similar conclusion to ours [34].

A few assessments for T&Y, for example, Pediatric Modified Dynamic Gait Index [35] and Functional Gait Assessment modified for children [36], include head turns when walking, which is often demanding when having a vestibular dysfunction. In the mentioned tests, the head turns are not as fast and frequent as desirable, and they are not evaluated separately but together with the whole assessment. Our intention was therefore to explore whether the separate items from “Walking test with or without head movement” could be appropriate to distinguish vestibular function in a younger cohort population, including T&Y. The Walking 10-m, Self-selected speed, head turns, points, was the item with the highest statistically significant difference between the groups (NVF vs. VL and subgroups CI-BVI vs. NH and CI-NVF). However, from a clinical perspective, the 1-point difference in median points on the tests between groups was too small to be clinically useful, indicating a ceiling effect due to that the test was not challenging enough. Another explanation for similar point scores between groups could be that the TAYACI had compensated for their vestibular impairment.

### How did CI presence and activity (on vs. off) affect balance?

In our study, similar balance was observed in NH and TAYACI with CI on and off across all items, aligning with studies by Suarez [15] and Huang [14] that have demonstrated no difference in balance capacity with an active CI. Our findings therefore did not support research suggesting that CI might mitigate the effects of vestibular impairment, for example, enhancing balance through electrical spread or by a better spatial orientation given by hearing cues.

### How physically active was TAYACI?

Surprisingly, no significant difference in physical activity level was observed between TAYACI, NH, and vestibular subgroups using the SGPALS, although the distribution across groups was unequal. The majority of TAYACI were engaged in regular physical training, while most of the NH participated at the most physically challenging level. It is possible that the sample size was too small to detect a significant difference. To our knowledge, SGPALS has not been previously used with TAYACI.

There was also no difference in participation in physical education in school between the groups, where the largest part in all groups reported that they always attended, and none reported that they never attended. We are not aware of any studies where T&Y have been asked about how they experience their balance and physical activity. In our opinion, it is essential not only to determine how the balance is affected, but also to understand how the TAYACI cope with activities where a balance impairment might impact them and, in the worst case, lead to inactivity and avoidance. A study by Cushing et al. [16] asked the parents about their children’s recreational activities. The parents often stated that their children found activities such as biking and skating difficult and that the children, for example, required training wheels for a prolonged time.

It was gratifying that TAYACI, according to the responses from the free text question “What activities do you perform in your spare time?”, participated in exercises such as ball sports and racket sports, which can be demanding as they involve quick head and body movements. To our knowledge, one study [37] has queried TAYACI about sports activities, and their results aligned with our findings that a large portion was involved in sports activities. TAYACI also did not avoid more activities than NH in physical education at school. The most common reason for avoiding activities for TAYACI was issues related to the CI. They were concerned about breaking the CI, that it would be visible to others while running, having difficulties in hearing instructions during swimming or being required to have a sign interpreter present during swimming, which they found embarrassing.

The results above were gladdening since 40% of TAYACI found their balance inferior to others, and, nevertheless, they had an active lifestyle with physical activity. Despite difficulties with balance, more prominent in static balance test from BOT2, TAYACI were not limited in physical activities. In Kids BESTest Reactive Postural Responses, where balance reactions were tested, the CI-UVI and CI-BVI showed good balance reactions similar to the results in NH. This might be a reason why they, unlike children with neurological disorders in general, could compensate for the vestibular impairment and were not hindered.

### Strengths and limitations

There are several limitations in the study with respect to subgroup analysis and balance tests chosen. With respect to subgroup analysis, the sample size was relatively small, resulting in uneven distribution, especially in the UVI group, limiting the generalisation of results. Another limitation is the consequence of using instruments measuring balance developed mainly for use in a population including individuals with neurological conditions. Thereby, the tasks tested might not have been challenging enough to detect a vestibular impairment, leading to a ceiling effect in some of the items when used in our cohort. The marginal but significant difference in age distribution between the tested population and the reference group 1.2 years older (*p* = 0.04) is, in our opinion, of minor concern considering the age span for inclusion (12–22 years of age). However, a strength of the study is the use of standardised instruments developed for use in a paediatric population. In addition, the tests are often used both in a clinical setting and for research, enhancing comparisons of results with others. Another strength is that about 50% of the TAYACI had NVF, making it possible to analyse and conclude that it is the vestibular impairment and not the CI that affects the balance.

## Conclusions

Vestibular impairment was common among teenagers and young adults with CI. Results from balance tests showed reduced capacity that was more evident in teenagers and young adults with vestibular impairment. However, they effectively compensated for their functional limitations and exhibited levels of physical activity similar to their peers with normal hearing. We suggest sensitive items to screen vestibular impairment in standard care; namely, items 4, 6, and 9 from the balance subtest on BOT-2, where BOT9 testing standing on one leg on a balance beam with eyes closed, had the best properties for screening. Nonetheless, none of the tests in this study could differentiate between uni- and bilateral vestibular impairment, nor between teenagers and young adults with CI and normal vestibular function and peers with normal hearing. Balance capacity was not influenced by the condition of CI turned on or off. This study adds new knowledge to the limited existing literature on balance capacity and physical activity in teenagers and young adults who received CI in early childhood. The results can be generalised to teenagers and young adults irrespective of having CI, since vestibular impairment, rather than CI, caused balance difficulties. A quick-to-perform and accessible balance test can be integrated into.

paediatric standard care to identify young individuals with vestibular impairment, regardless of their balance in daily life. Finally, performing less than 3 s on BOT9 may raise the suspicion of vestibular impairment in the absence of other known factors.

## Supplementary Information

Supplementary Material 1.

Supplementary Material 2.

Supplementary Material 3.

## Data Availability

The datasets analysed during the current study are available from the corresponding author upon request.

## Abbreviations

UVI: Unilateral Vestibular Impairment
BVI: Bilateral Vestibular Impairment
CI: Cochlear Implant
HRQoL: Health Related Quality of Life
T&Y: Teenagers and Young Adults
TAYACI: Teenagers and Young Adults with Cochlear Implants
NH: Teenagers and Young Adults with Normal Hearing
vHIT: video Head Impulse Test
VOR: Vestibulo-Ocular-Reflex
aVOR: Angular Vestibulo-Ocular-Reflex
BOT-2: Bruininks-Oseretsky Test of Motor Proficiency Second Edition
Kids-BESTest: Kids-Balance Evaluation System Test
SGPALS: Saltin-Grimby Physical Activity Level Scale
NVF: Total sample with Normal Vestibular Function (regardless of having CI or not)
VL: TAYACI with Vestibular Impairment (unilateral and bilateral)
CI-UVI: Subgroup of TAYACI with Unilateral Vestibular Impairment
CI-BVI: Subgroup of TAYACI with Bilateral Vestibular Impairment
CI-NVF: Subgroup of TAYACI with Normal Vestibular Function
