# Supplementary material for: Balance and physical activity in teenagers and young adults with cochlear implants: a Swedish cohort study
Source: BMC Pediatr. 2026 Mar 5;26:278. doi: 10.1186/s12887-026-06669-x (PMC13064083; doi:10.1186/s12887-026-06669-x)
Supplement: Supplementary file 1 — Supplementary Material 1. [file 12887_2026_6669_MOESM1_ESM.pptx]

## Slide 1
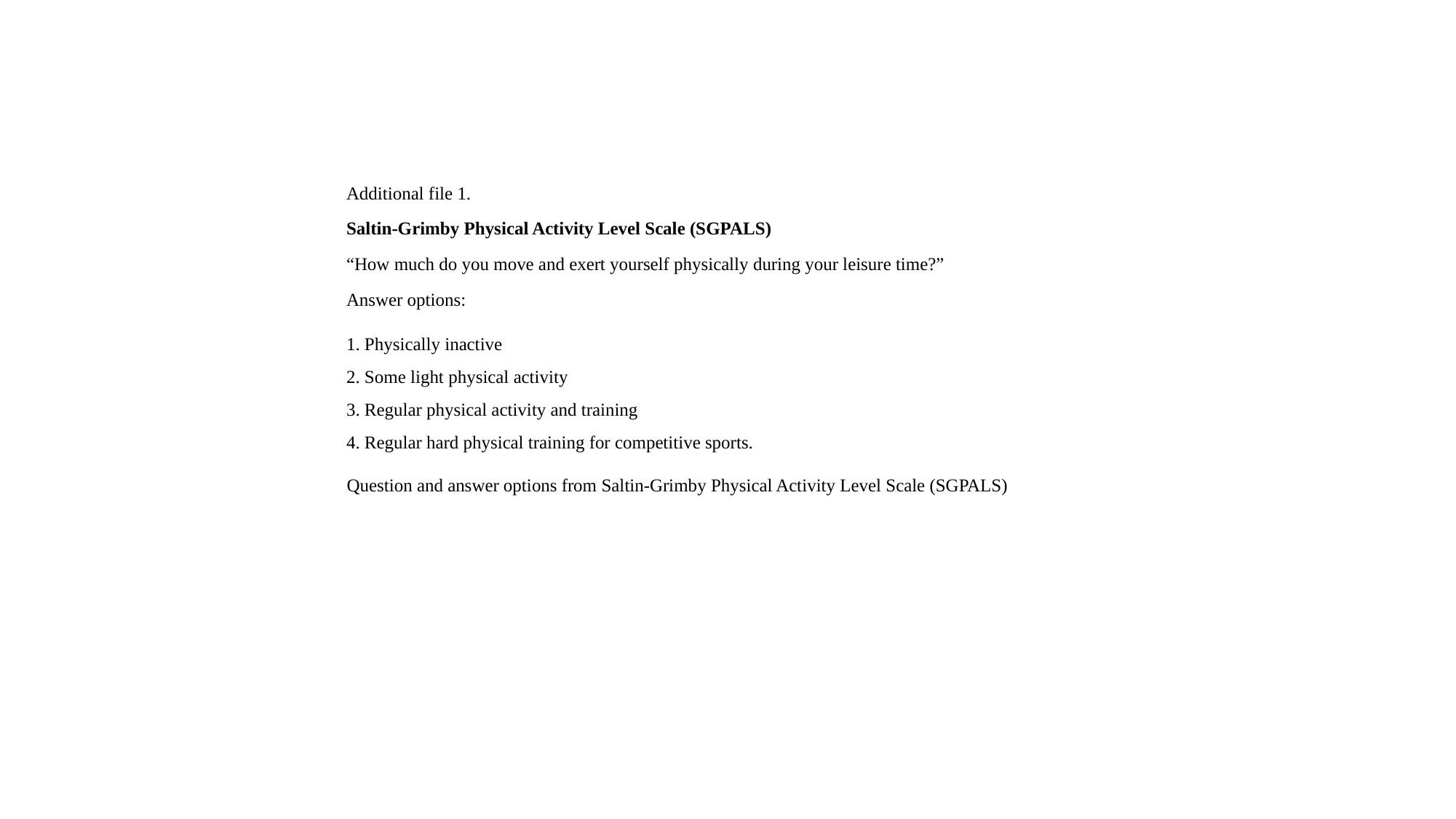

Additional file 1.
Saltin-Grimby Physical Activity Level Scale (SGPALS)
“How much do you move and exert yourself physically during your leisure time?”
Answer options:
1. Physically inactive
2. Some light physical activity
3. Regular physical activity and training
4. Regular hard physical training for competitive sports.
Question and answer options from Saltin-Grimby Physical Activity Level Scale (SGPALS)

## Slide 2
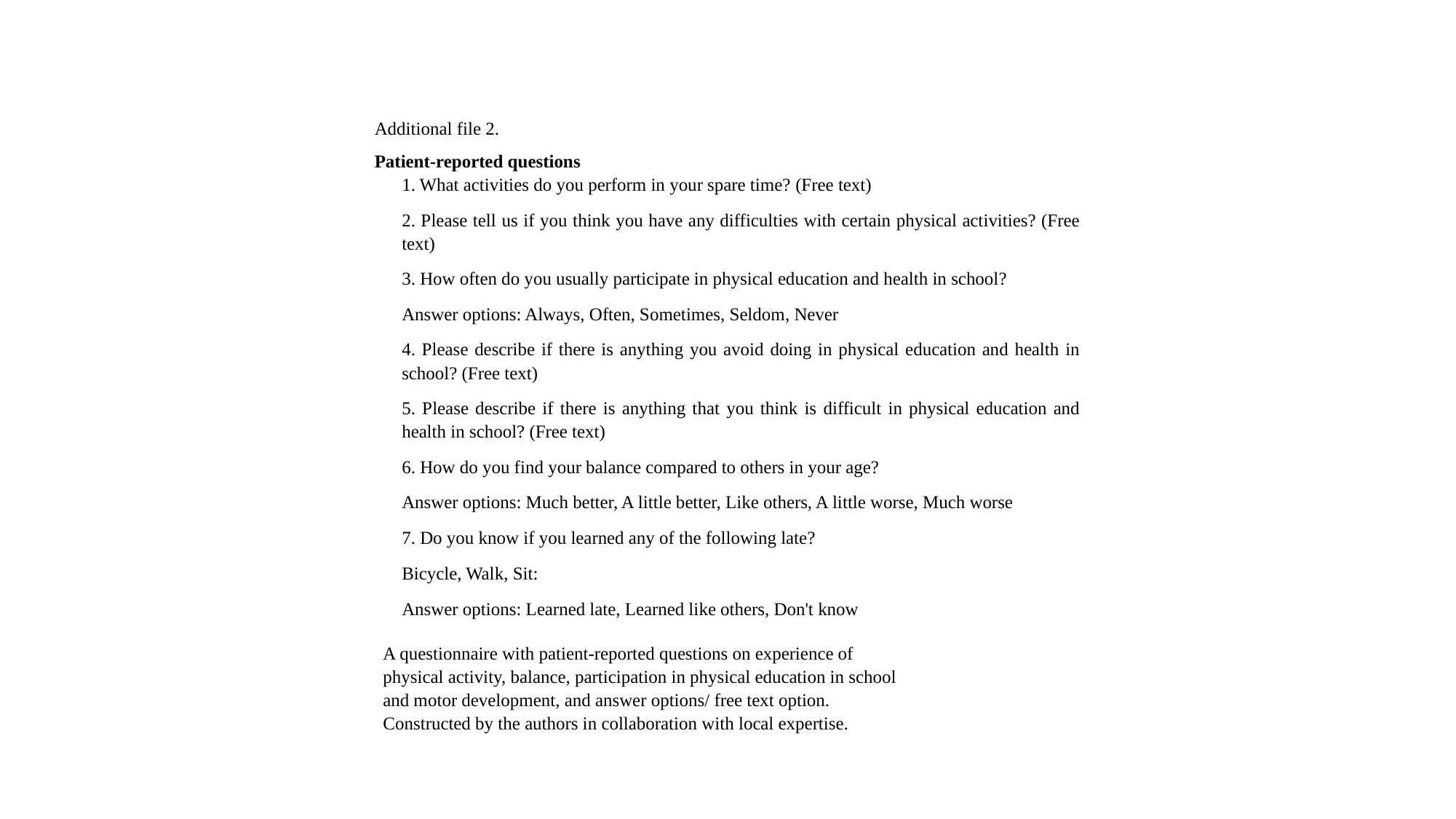

Additional file 2.
Patient-reported questions
1. What activities do you perform in your spare time? (Free text)
2. Please tell us if you think you have any difficulties with certain physical activities? (Free text)
3. How often do you usually participate in physical education and health in school?
Answer options: Always, Often, Sometimes, Seldom, Never
4. Please describe if there is anything you avoid doing in physical education and health in school? (Free text)
5. Please describe if there is anything that you think is difficult in physical education and health in school? (Free text)
6. How do you find your balance compared to others in your age?
Answer options: Much better, A little better, Like others, A little worse, Much worse
7. Do you know if you learned any of the following late?
Bicycle, Walk, Sit:
Answer options: Learned late, Learned like others, Don't know
A questionnaire with patient-reported questions on experience of physical activity, balance, participation in physical education in school and motor development, and answer options/ free text option. Constructed by the authors in collaboration with local expertise.

## Slide 3
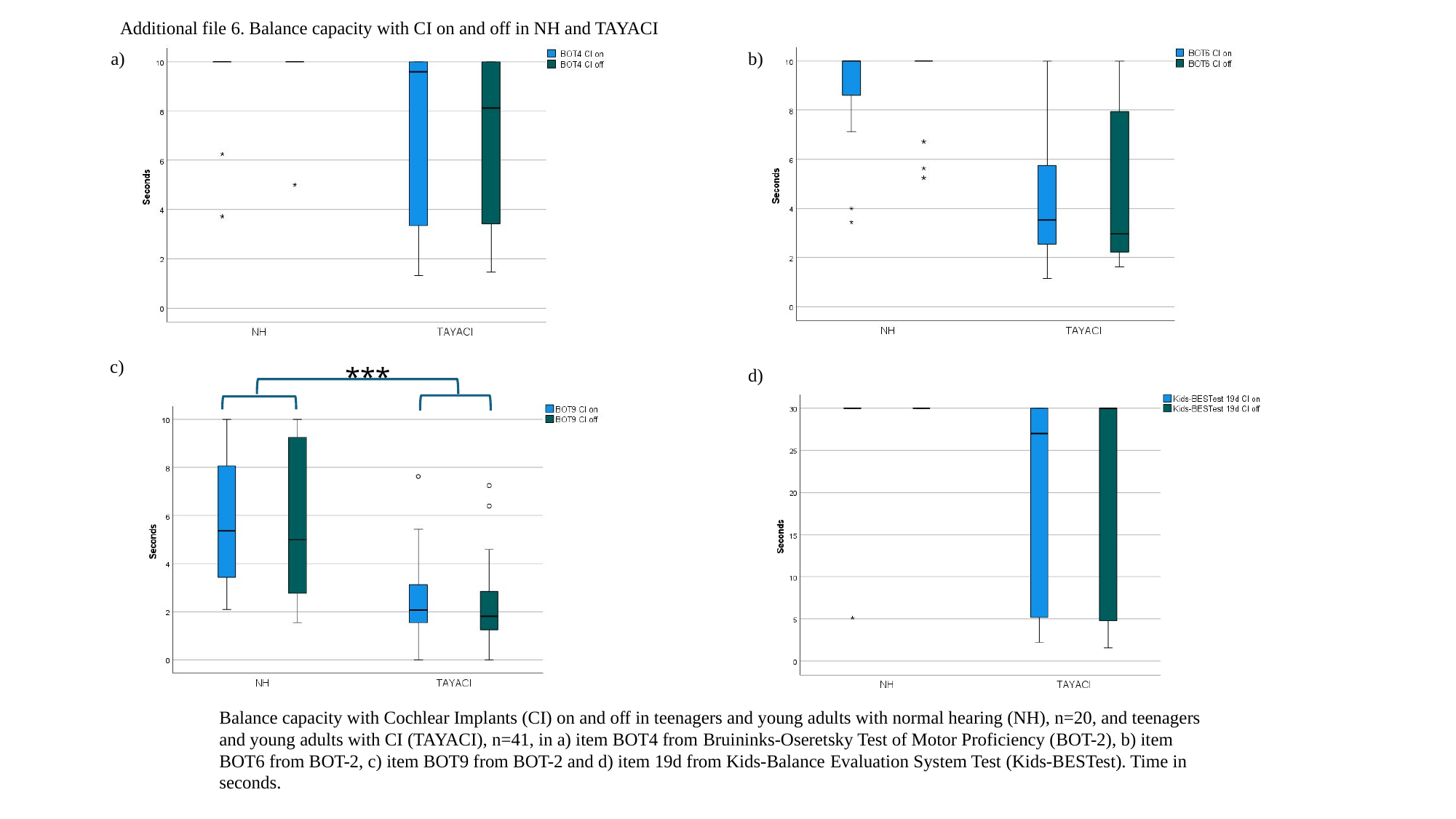

Additional file 6. Balance capacity with CI on and off in NH and TAYACI
a)
b)
c)
***
d)
Balance capacity with Cochlear Implants (CI) on and off in teenagers and young adults with normal hearing (NH), n=20, and teenagers and young adults with CI (TAYACI), n=41, in a) item BOT4 from Bruininks-Oseretsky Test of Motor Proficiency (BOT-2), b) item BOT6 from BOT-2, c) item BOT9 from BOT-2 and d) item 19d from Kids-Balance Evaluation System Test (Kids-BESTest). Time in seconds.

## Slide 4
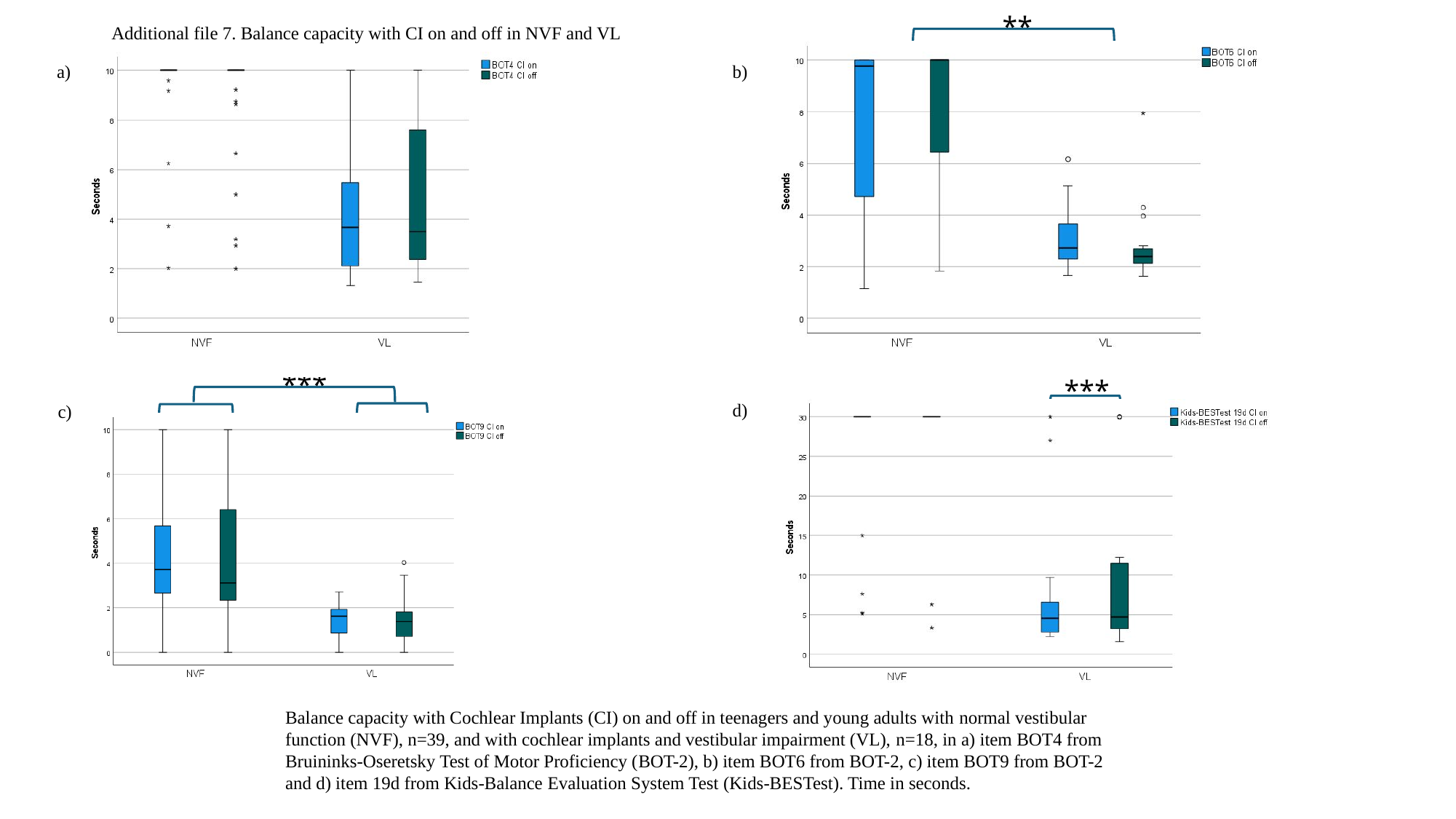

**
Additional file 7. Balance capacity with CI on and off in NVF and VL
a)
b)
***
***
d)
c)
Balance capacity with Cochlear Implants (CI) on and off in teenagers and young adults with normal vestibular function (NVF), n=39, and with cochlear implants and vestibular impairment (VL), n=18, in a) item BOT4 from Bruininks-Oseretsky Test of Motor Proficiency (BOT-2), b) item BOT6 from BOT-2, c) item BOT9 from BOT-2 and d) item 19d from Kids-Balance Evaluation System Test (Kids-BESTest). Time in seconds.

## Slide 5
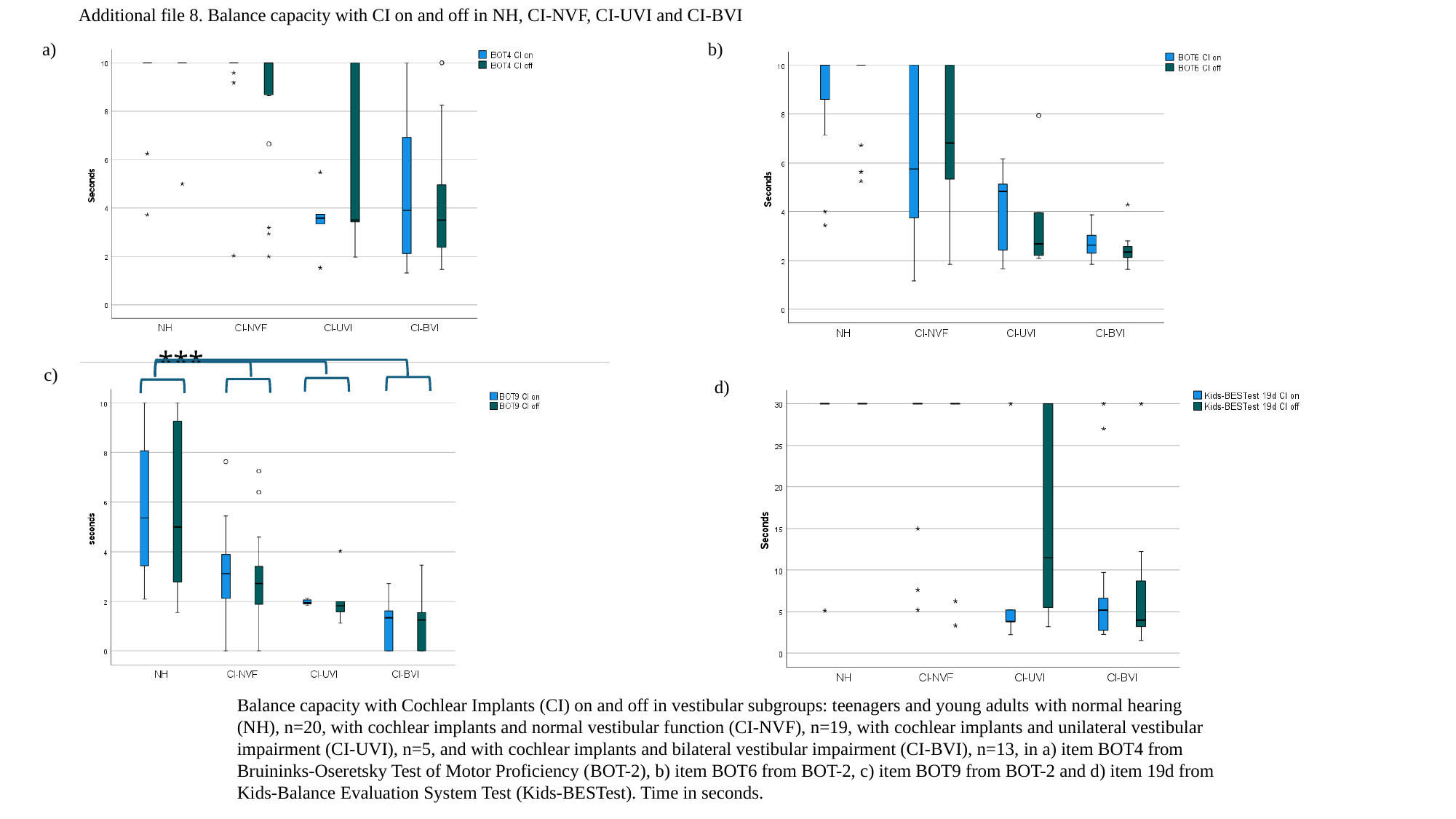

Additional file 8. Balance capacity with CI on and off in NH, CI-NVF, CI-UVI and CI-BVI
a)
b)
***
c)
d)
Balance capacity with Cochlear Implants (CI) on and off in vestibular subgroups: teenagers and young adults with normal hearing (NH), n=20, with cochlear implants and normal vestibular function (CI-NVF), n=19, with cochlear implants and unilateral vestibular impairment (CI-UVI), n=5, and with cochlear implants and bilateral vestibular impairment (CI-BVI), n=13, in a) item BOT4 from Bruininks-Oseretsky Test of Motor Proficiency (BOT-2), b) item BOT6 from BOT-2, c) item BOT9 from BOT-2 and d) item 19d from Kids-Balance Evaluation System Test (Kids-BESTest). Time in seconds.
